# Upconversion-mediated ZnFe_2_O_4_ nanoplatform for NIR-enhanced chemodynamic and photodynamic therapy†
†Electronic supplementary information (ESI) available: XPS spectra of typical elements Zn 2p; FT-IR spectra of the DA/Y-UCSZ and DOX loaded PEG/Y-UCSZ samples; the changes of zeta potentials for nanoparticles obtained at each PEGylated step; decay curves for ^1^G_4_–^3^H_6_ emission (475 nm) of Tm^3+^ in UCS, Y-UCSZ; EPR spectra of ^1^O_2_ and ˙OH in the PEG/Y-UCSZ aqueous solution; DOX release efficiency from PEG/Y-UCSZ&DOX in PBS at varied temperatures and pH values; the hemolytic percentage of PEG/Y-UCSZ in human red blood cells; H&E stained images of liver, lung, kidney, heart and spleen obtained from different groups after 14 days treatment. See DOI: 10.1039/c9sc00387h


**DOI:** 10.1039/c9sc00387h

**Published:** 2019-03-06

**Authors:** Shuming Dong, Jiating Xu, Tao Jia, Mengshu Xu, Chongna Zhong, Guixin Yang, Jiarong Li, Dan Yang, Fei He, Shili Gai, Piaoping Yang, Jun Lin

**Affiliations:** a Key Laboratory of Superlight Materials and Surface Technology , Ministry of Education , College of Materials Science and Chemical Engineering , Harbin Engineering University , Harbin , 150001 , P. R. China . Email: gaishili@hrbeu.edu.cn ; Email: yangpiaoping@hrbeu.edu.cn; b State Key Laboratory of Rare Earth Resource Utilization , Changchun Institute of Applied Chemistry , Chinese Academy of Sciences , Changchun 130021 , P. R. China . Email: jlin@ciac.ac.cn

## Abstract

ZnFe_2_O_4_, a semiconductor catalyst with high photocatalytic activity, is ultrasensitive to ultraviolet (UV) light and tumor H_2_O_2_ for producing reactive oxygen species (ROS). Thereby, ZnFe_2_O_4_ can be used for photodynamic therapy (PDT) from direct electron transfer and the newly defined chemodynamic therapy (CDT) from the Fenton reaction. However, UV light has confined applicability because of its high phototoxicity, low penetration, and speedy attenuation in the biotissue. Herein, an upconversion-mediated nanoplatform with a mesoporous ZnFe_2_O_4_ shell was developed for near-infrared (NIR) light enhanced CDT and PDT. The nanoplatform (denoted as Y-UCSZ) was comprised of upconversion nanoparticles (UCNPs), silica shell, and mesoporous ZnFe_2_O_4_ shell and was synthesized through a facile hydrothermal method. The UCNPs can efficiently transfer penetrable NIR photons to UV light, which can activate ZnFe_2_O_4_ for producing singlet oxygen thus promoting the Fenton reaction for ROS generation. Besides, Y-UCSZ possesses enormous internal space, which is highly beneficial for housing DOX (doxorubicin, a chemotherapeutic agent) to realize chemotherapy. Moreover, the *T*_2_-weighted magnetic resonance imaging (MRI) effect from Fe^3+^ and Gd^3+^ ions in combination with the inherent upconversion luminescence (UCL) imaging and computed tomography (CT) from the UCNPs makes an all-in-one diagnosis and treatment system. Importantly, *in vitro* and *in vivo* assays authenticated excellent biocompatibility of the PEGylated Y-UCSZ (PEG/Y-UCSZ) and high anticancer effectiveness of the DOX loaded PEG/Y-UCSZ (PEG/Y-UCSZ&DOX), indicating its potential application in the cancer treatment field.

## Abstract 2

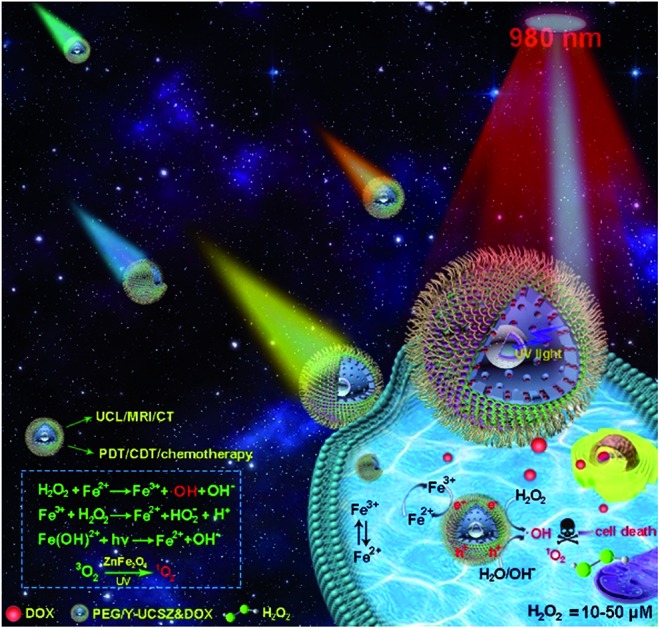
ZnFe_2_O_4_, a semiconductor catalyst with high photocatalytic activity, is ultrasensitive to ultraviolet (UV) light and tumor H_2_O_2_ for producing reactive oxygen species (ROS).

## Introduction

Cancer is one of the greatest menaces to human health, which causes millions of deaths worldwide each year.^1^–^5^ To cope with cancer, innovations of intelligent nanoplatforms with both therapeutic and diagnostic functionalities are urgently desired. Photodynamic therapy (PDT) is the most potential therapeutic strategy for various types of cancers, which involves a generation of hypertoxic reactive oxygen species (ROS) upon activation with a specific wavelength of photosensitizer-activating light.^6^–^13^ As recognized, PDT has several outstanding advantages compared with other conventional treatments, such as minimal trauma, high selectivity and favorable safety. However, some unresolved challenges have severely impeded further clinical applications of PDT. First of all, the conventionally used organic photosensitizers have low water solubility and poor stability, which often induce high toxicity to normal tissue. Secondly, low photodynamic efficiency leads to an insufficient treatment effect. Finally, the low penetration depth of UV-Vis light leads to incapability of reaching the deep pathological tissues or organs.^14^–^17^ Therefore, various innovative methods have been developed to improve the efficiency of the treatment.

The newly defined chemodynamic therapy (CDT) may be a good method to enhance the efficiency of PDT, especially when mediated by a special material which can realize both therapies in a tumor microenvironment.^18^,^19^ It is known that solid tumors can produce abundant H_2_O_2_ (from 10 to 50 μM) because of the abnormal metabolism of tumor cells.^20^–^23^ Accordingly, CDT will employ the iron-initiated Fenton reaction to induce the apoptosis of tumor cells by transferring endogenous H_2_O_2_ into the highly toxic hydroxyl radical (˙OH). Thus, integration of PDT and CDT from the Fenton reaction to develop a ROS-mediated therapeutic nanoplatform is a promising method for enhancing the antitumor efficacy. Importantly, the irradiation of UV light has been acknowledged as a feasible strategy to improve the Fenton reaction.^24^–^26^

Among various materials, the spinel ferrite was chosen in this work on account of its narrow band gap, high stability, magnetic properties and promise for both PDT and CDT agents. Many research groups have done a lot of work on ferrite for cancer treatment.^27^–^32^ Recently, Zhang and co-workers successfully prepared copper ferrite nanoparticles with enhanced ROS production in the presence of a 650 nm laser.^14^ However, biotissues have higher absorptivity in the shorter-wavelength-light range, thus the 650 nm light has intrinsic drawbacks including limited tissue penetration depth and potential overheating issues. Similarly, for the UV-light-excited spinel ferrite, non-negligible problems of lower penetration depth, potential tissue damage and speedy attenuation still impose restrictions on its application in treating deep-seated tumor below the skin. In other words, it's urgent to find other light sources. Alternatively, it was revealed that near-infrared (NIR) light lying in the biological window (700–1000 nm) has the distinctive advantages of deep photon penetration, enhanced image contrast, and minimal damage to living organisms, all of which are quite essential for biomedical applications.^33^–^38^ Fortunately, lanthanide-doped upconversion nanoparticles (UCNPs) have made tremendous progress in photon upconversion, which provides an alternative way to transform longer wavelength NIR laser into shorter wavelength light (UV or Vis light).^39^–^45^ Thus, Yb^3+^/Tm^3+^-codoped UCNPs have been constructed to serve as an effective UV-Vis source in this article.^46^–^48^ Besides, Gd^3+^/Yb^3+^ co-doped nanoparticles have been certified as superb CT contrast agents thanks to their high atomic number and the strong X-ray attenuation of the dopants, which are of great significance for cancer diagnosis.^49^–^52^

Based on these analyses, for the first time, we have developed a multifunctional theranostic platform based on a yolk mesoporous nanostructure (Y-UCSZ) for multimodal therapy (PDT, CDT and chemotherapy) under the guidance of trimodal imaging, so as to attain tumor-specific enhanced antitumor efficacy and diagnosis. In this system, the NIR-excited and Yb^3+^/Tm^3+^-codoped UCNPs served as the UV-Vis source, in the place of direct UV radiation. The coated mesoporous ZnFe_2_O_4_ shell with a redox pair (Fe^2+^/Fe^3+^) can efficiently produce more virulent ˙OH through the Fenton reaction after absorbing UV light, realizing an excellent photo-enhanced CDT.^14^,^26^ As an effective photocatalyst, on the one hand, cytotoxic ˙OH can be produced by a photogenerated electron/hole pair of ZnFe_2_O_4_.^53^–^57^ On the other hand, the ZnFe_2_O_4_ nanoparticles converted oxygen into highly toxic ^1^O_2_ after absorbing UV light energy upon the irradiation of NIR light. Meanwhile, the yolk nanostructures with mesoporous shells and large cavities provide an excellent amount of DOX loading. And the nanocarriers can release DOX in response to the tumor microenvironment (chemotherapy), further enhancing the antitumor efficacy. For further biological applications, PEGylation endows the Y-UCSZ nanotheranostics with high biocompatibility, making them selectively accumulate in tumor regions *via* the enhanced permeability and retention effect (EPR).^58^,^59^ Hence, a superb *in vivo* synergistic therapeutic effect is then achieved after incorporating chemotherapy with PDT and CDT under the imaging-guidance (UCL, MRI, CT), suggesting its promising clinical application under the accurate observation of nanoparticles in tumor sites.

## Results and discussion

**Scheme 1:**
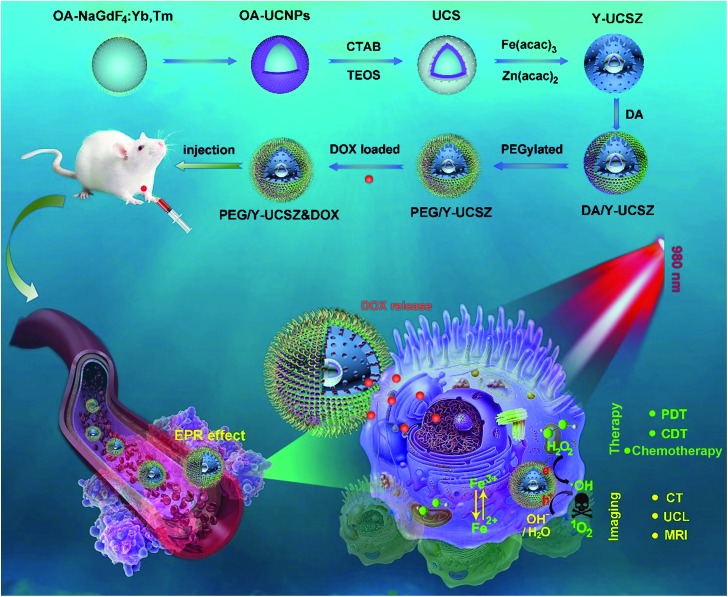
Schematic illustration of the synthesis of PEG/Y-UCSZ&DOX, and the transport of DOX-loaded PEG/Y-UCSZ in the blood vessel, enhanced trimodal (PDT/CDT/chemotherapy) therapy and multiple bioimaging.

The formation process of the PEG/Y-UCSZ&DOX sample and trimodal treatment process with imaging-guidance are depicted in Scheme 1. As displayed, there are several imperative steps in the synthetic process. Firstly, the NaGdF_4_:Yb,Tm core nanoparticles were prepared by a high-temperature decomposition method.^60^ Afterwards, the active-shell (NaGdF_4_:Yb) was covered on the surface of NaGdF_4_:Yb,Tm to acquire the UCNPs *via* an epitaxial growth method, which is conducive to achieve enhanced upconversion of NaGdF_4_:Yb,Tm core nanoparticles upon the irradiation of a 980 nm laser.^61^–^63^ A layer of mesoporous silica (mSiO_2_) was grown on UCNPs further. Importantly, some researchers found that the surface properties and catalytic performance of the materials could be modified by adjusting the structures of materials. Yolk nanostructures with mesopores possess a larger specific surface area, which is beneficial to the formation of more reactive sites for PDT and CDT.^53^,^56^ Hence, a gas–liquid template method was used to enable the formation of yolk-like upconversion nanostructures UCNPs@mSiO_2_@Y-ZnFe_2_O_4_ (abbreviated as Y-UCSZ in this work), owing to the decomposition of carboxylate under hydrothermal conditions into gaseous species including CO_2_. After that, dopamine (DA) and mPEG–COOH were employed in turns to modify the surface of Y-UCSZ in order to potentiate the biocompatibility of PEG/Y-UCSZ and alleviate the immunogenicity of the host's immune system. Through these elaborative designs, a multifunctional therapeutic nanosystem with superior multiple imaging-guidance is obtained. After that, the nanosized PEG/Y-UCSZ&DOX was injected *in situ* and transported inside blood vessels, and selectively accumulated at tumor sites through the EPR effect. When irradiated by the 980 nm light, the PEG/Y-UCSZ&DOX particles can produce toxic ROS by PDT and CDT, arousing the apoptosis of tumor cells. And the DOX can be released from the carriers to achieve chemotherapeutic performance. In a word, the PEG/Y-UCSZ-DOX nanosystem is greatly promising in realizing enhanced antitumor efficacy and the accurate diagnosis of tumor.

**Fig. 1:**
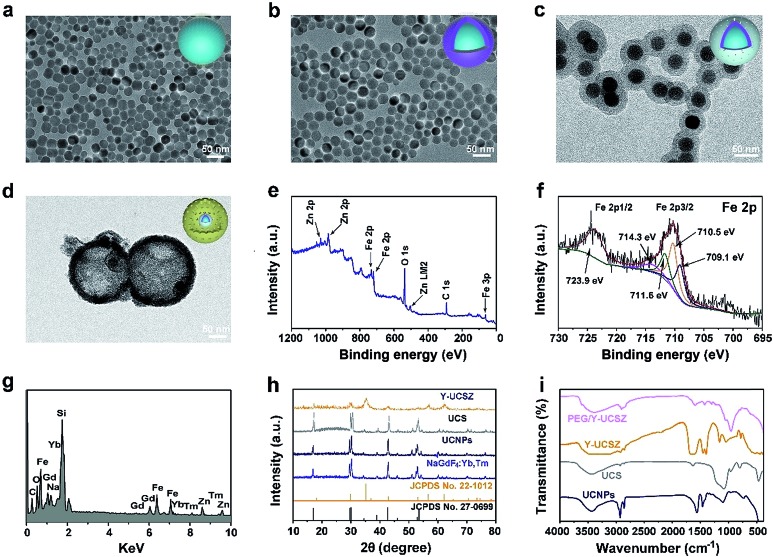
TEM images of NaGdF_4_:Yb,Tm (a), core–shell UCNPs (b), UCS (c) and Y-UCSZ (d); XPS spectra of Y-UCSZ (e) and typical elemental Fe 2p in Y-UCSZ treated with H_2_O_2_ (f), EDS spectrum of Y-UCSZ (g), XRD patterns (the standard patterns of β-NaGdF_4_ and ZnFe_2_O_4_ are given for comparison) (h), and FT-IR spectra (i) of the as-prepared UCNPs, UCS, Y-UCSZ and PEG/Y-UCSZ nanoparticles.

Fig. 1a–d exhibit the TEM photographs of β-NaGdF_4_:Yb,Tm, UCNPs, UCS and Y-UCSZ, respectively. As shown in Fig. 1a, the uniformity and monodispersity of NaGdF_4_:Yb,Tm particles were well maintained (mean diameter: 20 nm). Fig. 1b shows the TEM image of UCNPs, and the sample consists of discrete and uniform nanoparticles, and the average size is about 29.1 nm. Before the mesoporous silica shell coating, CTAB was used to modify UCNPs so as to obtain hydrophilic nanoparticles. The TEM photograph presented in Fig. 1c shows that the UCNPs@mSiO_2_ particles maintain good dispersity, and the size of UCPZ-PEG is about 42.1 nm. Afterward, a mesoporous ZnFe_2_O_4_ shell was further coated on the SiO_2_ shell through a facile hydrothermal method to obtain the yolk-structured nanoparticles (Y-UCSZ) with a mean size of 178.5 nm (Fig. 1d). X-ray photoelectron spectra (XPS) measurement was carried out to investigate the basic elements. The XPS measurement of Y-UCSZ in Fig. 1e demonstrates that the sample contains O, Zn and Fe elements, indicating that the ZnFe_2_O_4_ shell was coated on the UCS successfully. Fig. S1† exhibits two main peaks at 1022.4 and 1044.8 eV, which can be attributed to Zn 2p_3/2_ and Zn 2p_1/2_, respectively. Besides, the XPS spectra of Fe 2p treated with H_2_O_2_ are presented in Fig. 1f. The spectrum shows two main peaks at 723.9 and 710.5 eV, originating from Fe 2p_1/2_ and Fe 2p_3/2_ respectively, which are the typical oxidation states of iron in ZnFe_2_O_4_. Besides, there are four peaks at 709.1, 710.5, 711.6 and 714.3 eV, which can be assigned to Fe 2p_3/2_ after reacting with H_2_O_2_. According to a previous report, the peak at 709.1 eV could be attributed to Fe^2+^, and the other three peaks can be attributed to Fe^3+^. In particular, the ratio of Fe^2+^ and Fe^3+^ was calculated to be 0.21, indicating the occurrence of the Fenton reaction when treated with H_2_O_2_ and the radiation of 980 nm laser. Moreover, the EDS spectrum of Y-UCSZ in Fig. 1g describes the elemental composition of Y-UCSZ, directly certifying the successful synthesis of Y-UCSZ.

The XRD patterns of the NaGdF_4_:Yb,Tm, UCNPs, UCS, Y-UCSZ, the standard card of hexagonal β-NaGdF_4_ (JCPDS no. 27-0699) and ZnFe_2_O_4_ (JCPDS no. 22-1012) are displayed in Fig. 1h. The diffraction peaks of the as-fabricated NaGdF_4_:Yb,Tm and UCNP nanoparticles were consistent with the diffraction peaks of standard β-NaGdF_4_. In the XRD pattern of UCS, a new shoulder at 2*θ* = 22–28° apart from the representative peaks of UCNPs emerges from the silica coated on the UCNPs. Besides, the XRD pattern of Y-UCSZ can not only be fitted for the ZnFe_2_O_4_ phase (JCPDS no. 22-1012), but also be indexed to the normative β-NaGdF_4_ phase (JCPDS no. 27-0699), which demonstrates the successful synthesis of UCSZ.

The FT-IR spectra of UCNPs, UCS, Y-UCSZ and PEG/Y-UCSZ were observed to study the functional groups on the surface of nanoparticles, providing convincing results for the successful modification in every step. As displayed in Fig. 1i, the UCNPs capped with oleic acid display bands at 1463 and 1564 cm^–1^ associated with the vibrations of the carboxylic groups. And the intense transmission bands at 2854 and 2924 cm^–1^ derive from the asymmetric and symmetric stretching vibrations of –CH_2_. The peaks at 802 and 1088 cm^–1^ in the spectra of UCS are attributed to the vibration of Si–O–Si bands. After coating the ZnFe_2_O_4_ shell, the bands at 572 and 452 cm^–1^ can be assigned to the Fe–O and Zn–O bands, respectively.^56^ When the Y-UCSZ was modified with DA, an obvious peak at 1465 cm^–1^ in the spectrum is caused by the amino groups (–NH_2_–). The FT-IR spectrum of PEG/Y-UCSZ shows an obvious peak at 1638 cm^–1^, which is derived from the stretching vibrations of –CO–NH–. As for the PEG/Y-UCSZ&DOX sample, the new peaks at 1000–1800 cm^–1^ are caused by the loaded DOX (Fig. S2†). Zeta potentials of Y-UCSZ, DA/Y-UCSZ, and PEG/Y-UCSZ are exhibited in Fig. S3.† After reacting with DA, it can be obviously observed that the zeta potential changes from 4.62 to 20.3 mV, demonstrating the successful conjunction between DA molecules and Y-UCSZ nanoparticles. Afterwards, mPEG–COOH was utilized to connect the –NH_2_ from ZnFe_2_O_4_–DA with the corresponding potential of the samples transformed from 20.3 to –16.4 mV after PEGylation, implying that the surface modification is successfully achieved on the as-obtained Y-UCSZ. Besides, the size distributions of the samples in the different PEGylated steps were investigated by dynamic light scattering (DLS). As displayed in Fig. S4,† the sizes of the nanoparticles increase gradually in the process of PEGylation, demonstrating that the DA and mPEG–COOH connect to the Y-UCSZ nanoparticles successfully.

**Fig. 2:**
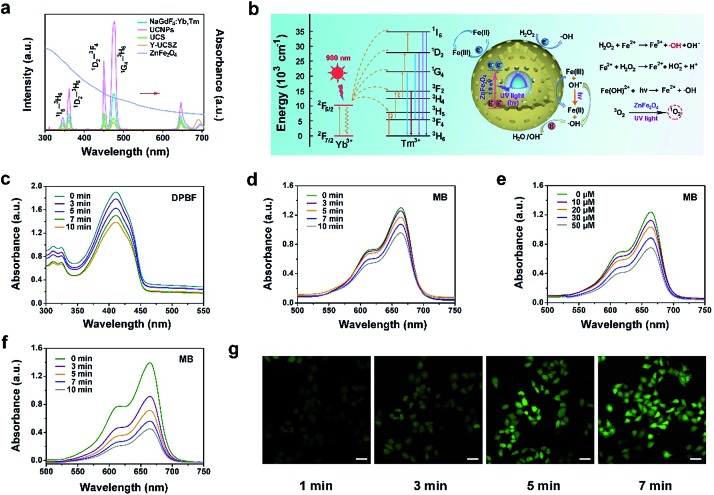
Absorption spectrum of ZnFe_2_O_4_ and emission spectra of β-NaGdF_4_:Yb,Tm, UCNPs, UCS, Y-UCSZ and UCPZ under the 980 nm laser excitation (a). Proposed energy transfer mechanism in the Yb^3+^/Tm^3+^ coupled Y-UCSZ and schematic of the separation and transfer of photo-generated electrons in the ZnFe_2_O_4_ nanoparticles combined with the possible Fenton reaction mechanism (b). The variation trend of the absorbance of DPBF mixed with the PEG/Y-UCSZ nanoparticles under 980 nm laser irradiation for different times (c). UV-Vis absorption spectra of MB after adding PEG/Y-UCSZ, upon designated time (*t* = 0–10 min) irradiation of a 980 nm laser (d), when reacted with different concentrations of H_2_O_2_ (e), and when reacted with H_2_O_2_ (50 μM) upon designated time (*t* = 0–10 min) irradiation of a 980 nm laser (f). Confocal laser scanning microscopy (CLSM) images of HeLa cells after incubating with PEG/Y-UCSZ under the 980 nm laser irradiation followed by reaction with DCFH-DA for 1, 3, 5 and 7 min (g). All laser pump powers are 0.8 W cm^–2^. Scale bar: 50 μm.

The upconversion emission spectra of β-NaGdF_4_:Yb,Tm, UCNPs, UCS, Y-UCSZ nanoparticles and UV-Vis absorption spectrum of ZnFe_2_O_4_ nanoparticles are shown in Fig. 2a. According to the theory, when combined with the energy receptor of the photosensitizer, the luminescent promoter would show a decline in the emission intensity. As displayed, there are apparent overlaps in the UV-Vis region of UCS and the absorbance peaks of ZnFe_2_O_4_. In particular, the UV-Vis emissions of UCNPs at 475 nm (^1^G_4_–^3^H_6_), 450 nm (^1^D_2_–^3^F_4_), 361 nm (^1^D_2_–^3^H_6_), and 345 nm (^1^I_6_–^3^H_6_) overlap with the UV-Vis absorption spectrum of ZnFe_2_O_4_. In comparison with UCS, after coating ZnFe_2_O_4_, the upconversion fluorescence intensity decreased remarkably in the UV-Vis region, implying that the UV radiation derived from UCNPs can be absorbed by the ZnFe_2_O_4_ shell to produce ^1^O_2_ and ˙OH. Besides, to further testify the energy transfer between UCS emission and Y-UCSZ upon the irradiation of a 980 nm laser, we investigated the emission decay curves of ^1^G_4_–^3^H_6_ (475 nm) in UCS and Y-UCSZ. As displayed in Fig. S5,† the markedly declined lifetime of Y-UCSZ compared to UCS (668.40 to 301.24 μs at 475 nm emission) demonstrates that the UV emission radiated from UCNPs can be efficiently absorbed by ZnFe_2_O_4_.

Hence, a key question whether the transferred energy could activate the ZnFe_2_O_4_ should be seriously investigated. In Fig. 2b, the relevant energy levels of Yb^3+^/Tm^3+^ coupled Y-UCSZ are exhibited to present the proposed energy transfer mechanism and the possible UV-Vis-driven reaction mechanisms for the production of ROS by ZnFe_2_O_4_ nanoparticles in the presence of UV-Vis light. When irradiated by UV-Vis light, electrons in the valence band (VB) of ZnFe_2_O_4_ can be photo-excited to the conduction band (CB) to produce electron–hole pairs. Then photogenerated holes (h^+^) in the VB of ZnFe_2_O_4_ can react with the surface-adsorbed H_2_O or OH^–^ to generate the highly reactive ˙OH. H_2_O_2_ can seize the electrons to generate ˙OH simultaneously. Besides, the Fe^3+^ on the ZnFe_2_O_4_ shell can react with electrons in the CB to obtain Fe^2+^, and the Fe^2+^ can be recovered from Fe^3+^ efficiently upon the radiation of a UV laser. As a result, more Fe^2+^ can react with H_2_O_2_ to generate more toxic ˙OH upon the irradiation of UV light. Meanwhile, the ZnFe_2_O_4_ nanoparticles converted oxygen into the highly toxic ^1^O_2_ after absorbing UV light energy. As displayed in Fig. 2c, the absorbance of DPBF solution mixed with PEG/Y-UCSZ under the 980 nm laser irradiation for diverse times decreases gradually as time goes on at the wavelength of 350–460 nm, indicating the efficient generation of ^1^O_2_. As a comparison, the absorption intensity of DPBF solution without PEG/Y-UCSZ just shows a slight decline under the 980 nm laser irradiation for 10 min (Fig. S6a†), which implies that the NIR contributes to some extent to the production of ^1^O_2_. Besides, we studied the ˙OH-generating activity of PEG/Y-UCSZ with or without NIR irradiation further. Methylene blue (MB), which can be degraded by ˙OH, was used to monitor the generation of ˙OH. As given in Fig. 2d and e, a distinct decrease of absorbance was noticed when MB was incubated with PEG/Y-UCSZ under 980 nm laser irradiation, and when incubated with PEG/Y-UCSZ and different concentrations of H_2_O_2_, which can be ascribed to the production of ˙OH. Notably, the decline rate of MB absorbance in Fig. 2f is faster than those in Fig. 2d and e, which indicate that the PEG/Y-UCSZ nanoparticles have higher ˙OH generation efficacy in the presence of H_2_O_2_ and NIR radiation, owing to the collaboration between PDT and photo-enhanced Fenton reaction. No obvious change in the MB absorbance was observed when the MB solution alone was treated with NIR radiation (Fig. S6b†), which illustrates the influence of the 980 nm laser. In addition, to further testify the generation of ROS, DCFH-DA, which could be oxidized to DCF with a green fluorescence upon irradiation of a 488 nm laser, was employed to detect intracellular ROS. Fig. 2g exhibits the CLSM images of HeLa cells cultured with PEG/Y-UCSZ for 1, 3, 5 and 7 min as well as after reaction with DCFH-DA upon the radiation of a 980 nm laser. The intensity of green fluorescence increases markedly, which also demonstrates the significant ROS production of PEG/Y-UCSZ with 980 nm laser irradiation.

Electron paramagnetic resonance (EPR) was performed to testify ^1^O_2_ and ˙OH produced by Y-UCSZ nanoparticles (Fig. S7a and b†), using TEMP (0.15 M) and DMPO (0.03 M). According to a previous report, TEMP can react with ^1^O_2_ to produce 2,2,6,6-tetramethyl-4-piperidone-*N*-oxyl and result in an EPR pattern which consists of three lines with equivalent intensity. As shown in Fig. S7a,† the EPR spectrum was in accordance with the above features, indicating the production of ^1^O_2_. Besides, DMPO can specifically capture ˙OH and form a DMPO–OH spin adduct further, thereby giving rise to four resolved peaks (Fig. S7b†).^64^,^65^

**Fig. 3:**
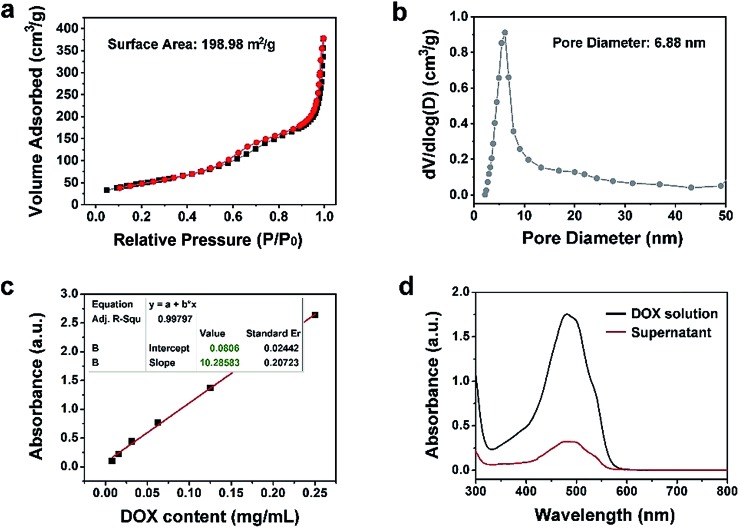
N_2_ absorption–desorption isotherm (a) and corresponding pore-size distribution of Y-UCSZ (b). The standard curve for DOX solutions detected at 480 nm (c). The absorbance spectra of the initial DOX solution and the supernatant obtained after the drug loading process with PEG/Y-UCSZ (d).

The N_2_ adsorption/desorption isotherm and the relevant pore-size distribution of Y-UCSZ are presented in Fig. 3a and b. As exhibited, Y-UCSZ shows a representative type IV isotherm, indicating the mesoporous structure of the nanoparticles. The Brunauer–Emmett–Teller (BET) surface area of the sample is measured to be 198.98 m^2^ g^–1^ with a high pore volume of 0.62 cm^3^ g^–1^ and corresponding average pore size of 6.88 nm. Notably, the nanoparticles with mesopores and large surface area are beneficial to load the chemotherapy drug DOX. Although the final sample (PEG/Y-UCSZ&DOX) still possesses a mesoporous structure, the BET surface area is reduced to 34.16 m^2^ g^–1^ coupled with a pore volume of 0.20 cm^3^ g^–1^ (Fig. S8a†), owing to that the DOX molecules are loaded into the mesoporous channels. Fig. 3c and d display the normative curve of DOX observed at 480 nm and the absorption spectra of the DOX solution before and after the loading procedure employing PEG/Y-UCSZ nanoparticles. Based on the Lambert–Beer law, the DOX loading rate of PEG/Y-UCSZ was calculated to be as high as 70.87% according to the standard curve and the absorbance intensity of DOX solution. Particularly, the UV-Vis absorption spectrum of PEG/Y-UCSZ&DOX has a distinct absorption peak consistent with the one of DOX in the range of 430 to 560 nm (Fig. S8b†), indicating that DOX is successfully loaded onto the nanoparticles. As reported, the tumor microenvironment has a higher temperature and lower pH value compared with normal tissue cells, which is caused by its abnormal metabolism. Herein, the tumor microenvironment-responsive release properties were investigated. As displayed in Fig. S9a,† PEG/Y-UCSZ&DOX shows a temperature-responsive drug release, exhibiting a positive increase with increasing temperature. The DOX release was also investigated by diluting PEG/Y-UCSZ&DOX in PBS with various pH values (Fig. S9b†). It can be noticed that the DOX release at 25 °C is about 8.6%, whereas the release rate respectively increases to 17.2% and 25.5% at temperatures of 37 and 50 °C. In addition, the drug release displays a much intensive effect in an acidic environment at 37 °C (Fig. S9c†). When the temperature is 37 °C and the pH value is 7.4, the release rate within 24 h is just 14.9%, while the release rate is as high as 36.6% at a pH value of 6.5, indicating that there is a distinct pH responsive release course.

**Fig. 4:**
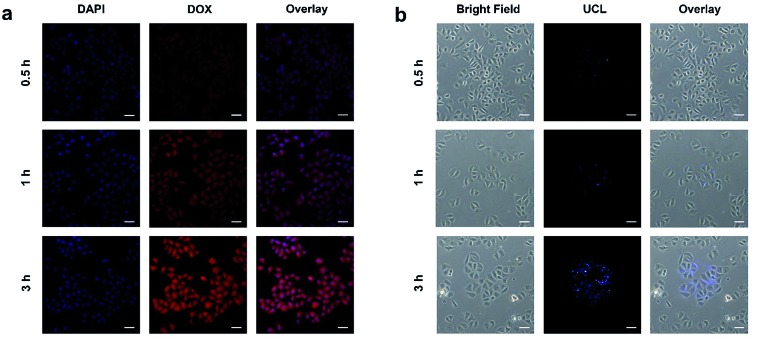
CLSM images of HeLa cells incubated with PEG/Y-UCSZ&DOX for 0.5, 1, and 3 h at 37 °C (a), and UCL microscopy images of HeLa cells incubated with PEG/Y-UCSZ NPs at 37 °C for 0.5, 1, and 3 h (b). Scale bar: 50 μm.

It is necessary to verify the cellular uptake behavior of the synthetic nanodrugs before *in vitro* anticancer application. Herein, the PEG/Y-UCSZ&DOX nanoparticles were added to the culture medium incubated with HeLa cancerous cells for 0.5, 1, and 3 h at 37 °C, and the corresponding CLSM photographs are shown in Fig. 4a. DAPI, an organic dye that can emit blue light in the presence of a 488 nm laser, was employed to mark the cell nuclei. And the DOX loaded in the nanoparticle radiates red emission upon the radiation of a 488 nm laser. Accordingly, the overlay photographs of the above two channels are exhibited. As displayed, there is only feeble red fluorescence in the first 0.5 h, demonstrating that only a small amount of PEG/Y-UCSZ&DOX has been swallowed by cells. With increase in incubation time, the intensity of the red signal becomes enhanced, indicating that more nanoparticles are located in the cells. The above results prove that the as-synthesized nanoparticles can be easily internalized by HeLa cells.

The upconversion luminescence radiated by PEG/Y-UCSZ nanoparticles could be employed to monitor the location of the nanodrug in the cells (Fig. 4b). To conceivably prove the phagocytosis of particles, PEG/Y-UCSZ nanoparticles were allowed to react with HeLa cells for 0.5, 1, and 3 h, respectively. As shown, the intensity of the blue emission shows positive enhancement with increase in the incubation time, which implies that the nanoparticles were swallowed by cells. In particular, the blue upconversion fluorescence is derived from PEG/Y-UCSZ NPs excited by the 980 nm laser. Significantly, most of the UCL signal is observed at the intracellular area, indicating that the nanoparticles have been internalized into the cells rather than just adhering to the surface of cell membranes. These results indicate that the PEG/Y-UCSZ nanoparticles are promising UCL imaging contrast agents with a negligible background.

**Fig. 5:**
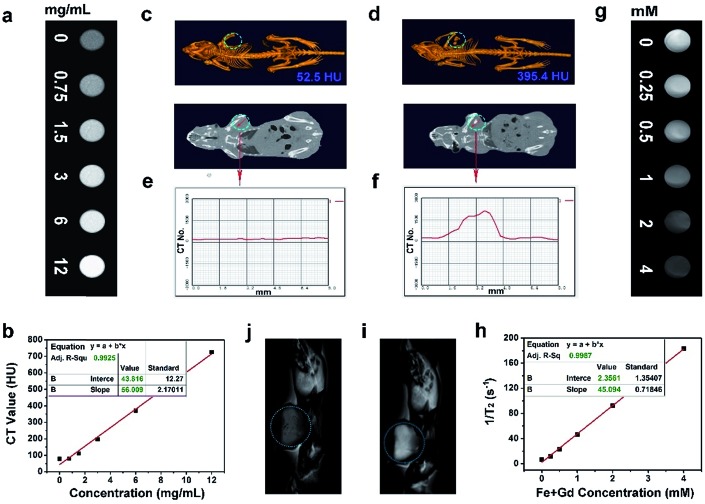
*In vitro* CT images with different PEG/Y-UCSZ concentrations (a), CT values of UCSZ aqueous solutions *versus* the PEG/Y-UCSZ concentrations (b). *In vivo* CT images of tumor-bearing mice without (c) and with (d) PEG/Y-UCSZ injection *in situ*, and the corresponding cross-sectional compositional line profiles of CT value pretreatment (e) and posttreatment (f). *In vitro T*_2_-weighted MR images of PEG/Y-UCSZ when incubated with PBS (g) and corresponding relaxation rate *r*_2_*versus* the total concentrations of Fe and Gd (h). *In vivo T*_2_-weighted MR images of tumor-bearing mice before (i) and after (j) injection of PEG/Y-UCSZ.

As accepted, the CT imaging technology can provide details of high-resolution three dimensional structures and deep tissue penetration. Besides, nanomaterials with lanthanide doping have been extensively studied for X-ray attenuation owing to the high atomic number of lanthanide elements. In this research, the *in vitro* and *in vivo* CT imaging properties of the Gd^3+^/Yb^3+^ doped PEG/Y-UCSZ sample were studied. As exhibited in Fig. 5a, when the concentrations of PEG/Y-UCSZ are boosted, the attenuation of X-ray increases markedly. Additionally, in Fig. 5b, the CT values show a linear dependence on sample concentrations, and the corresponding slope is calculated to be 56.009. To investigate the *in vivo* CT imaging performance of PEG/Y-UCSZ, the tumor-bearing mice without and with PEG/Y-UCSZ injection were imaged using a CT imaging instrument. Obviously, the tumor site without sample injection has a much lower CT value (52.5 HU, Fig. 5c) than the tumor site with injection (395.4 HU, Fig. 5d). Furthermore, the CT value profiles on the cross-sectional line of the tumor sites were investigated, and the corresponding results are displayed in Fig. 5e and f. Obviously, the CT value of the experimental group shows a fluctuation compared with the straight line of the control group. All of these results demonstrate that the PEG/Y-UCSZ nanoparticles can be employed as an efficient CT imaging contrast agent. According to previous reports, the Fe^3+^ and Gd^3+^ ions show positive enhancing capabilities of the *T*_2_ MRI signal,^26^,^66^ so we envisage that the integration of the ZnFe_2_O_4_ shell and the NaGdF_4_-based UCNPs can achieve a superior *T*_2_ MRI imaging outcome. Herein, the *T*_2_-weighted MRI effect of PEG/Y-UCSZ dispersed in PBS was investigated. As shown in Fig. 5g, the *T*_2_-weighted images demonstrate a clear concentration-dependent darkening efficacy. In Fig. 5h, the measured intensity of the *r*_2_ (1/*T*_2_) signal exhibits a linear increase with the total concentrations of Fe^3+^ and Gd^3+^ changing from 0 to 4 mM, with a high transverse relaxivity (*r*_2_) of 45.094 mM^–1^ s^–1^. In other words, the PEG/Y-UCSZ can generate the MR contrast on a transverse photon relaxation-time-weighted sequence to effectively shorten the *T*_2_ relaxation time. Afterwards, the *in vivo T*_2_-weighted MRI effect of PEG/Y-UCSZ nanoparticles was studied. As shown in Fig. 5i and j, there is an obvious *T*_2_-MR signal attenuation effect for the tumor with sample injection compared with the tumor without injection, indicating the great promise of PEG/Y-UCSZ as a *T*_2_ MRI contrast agent.

**Fig. 6:**
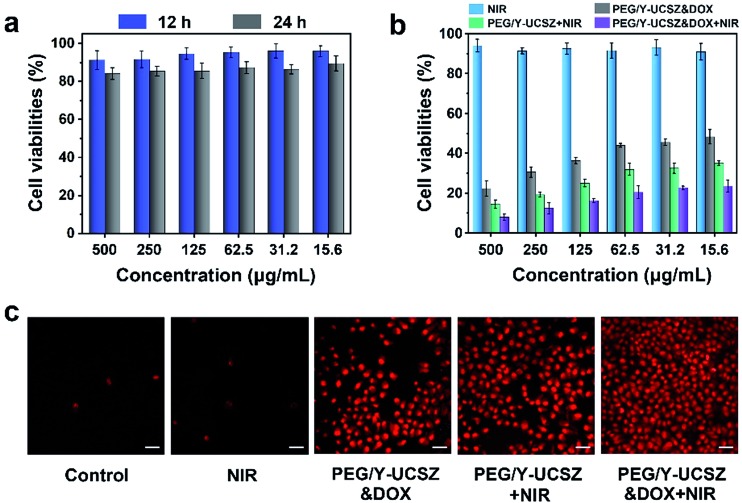
Viabilities of L929 fibroblast cells with incubation of PEG/Y-UCSZ for 12 and 24 h (a). Cytotoxicity of NIR, PEG/Y-UCSZ + NIR, PEG/Y-UCSZ&DOX and PEG/Y-UCSZ&DOX + NIR against HeLa cells (b). The CLSM images of HeLa cancer cells after various treatments, dyed with calcium PI. Scale bar: 50 μm (c).

As acknowledged, the biocompatibility of the as-obtained nanoparticles ought to be firstly evaluated before actual application. The viabilities of L929 cells after incubating with PEG/Y-UCSZ at different concentrations for 12 and 24 h are exhibited in Fig. 6a. The sample displays a high viability of 86.5–88.7% in the overall concentration limit even at 500 μg mL^–1^ after 24 h incubation, implying that the nanosystem has low toxicity. Besides, as displayed in Fig. 6b, the *in vitro* cytotoxicity of NIR, PEG/Y-UCSZ + NIR, PEG/Y-UCSZ&DOX and PEG/Y-UCSZ&DOX + NIR against HeLa cells was assessed by MTT measurement. In order to compare the anticancer effects of the obtained nanodrugs, the HeLa cells were subjected to different conditions and then cell viabilities were quantitatively measured employing the MTT method. The cell viability radiated with the 980 nm laser demonstrates that the 980 nm light displays no obvious toxicity to the cells. After PEG/Y-UCSZ incubation and then 980 nm laser irradiation, numerous HeLa cells are killed with clearly lower viability than that treated with NIR radiation only, owing to the coupling of PDT with a small Fenton effect. In addition, the viabilities of PEG/Y-UCSZ and NIR laser treated group are higher than those in the PEG/Y-UCSZ&DOX treated group, suggesting a PDT and photo-enhanced CDT effect. Obviously, the PEG/Y-UCSZ&DOX and 980 nm laser treated group shows the lowest cell viabilities, which indicates that the yolk-structured PEG/Y-UCSZ&DOX could actively collaborate PDT and CDT with chemotherapy to achieve the best therapeutic effect. In order to testify the cell-killing efficacy of various treatments, propidium iodide (PI), which can precisely dye dead cells with a red color, was used to differentiate cancerous cells (Fig. 6c). The results are well consistent with the above MTT anticancer assay. Especially in PEG/Y-UCSZ&DOX and NIR treated group, almost all cells are dead, implying the highest cancer killing effect of PEG/Y-UCSZ&DOX with NIR irradiation. Furthermore, the hemolysis results of PEG/Y-UCSZ are displayed in Fig. S10.† Inappreciable hemolysis can be observed with the highest hemolytic rate of about 5.03%, indicating mild toxicity to the normal cells of PEG/Y-UCSZ nanoparticles.

**Fig. 7:**
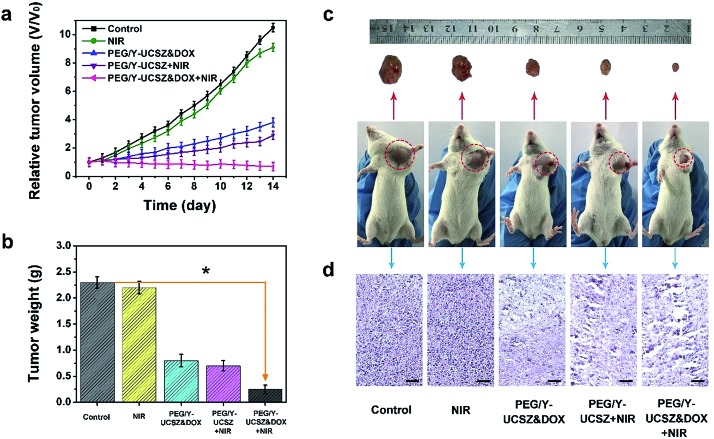
Changes in the relative tumor volume (a) and tumor weights (b) achieved for the mice with different treatments, *p < 0.01 *versus* control group. The data in (a) and (b) are the average calculated for five mice in each group. Photographs of representative mice and excised tumors (c). H&E stained images of tumor tissues obtained after 14 days therapy. Scale bar, 50 μm (d).

Inspired by the aforesaid *in vitro* therapy results, female mice were implanted with a U14 tumor xenograft and then subjected to *in vivo* therapeutic experiment. In particular, the tumor-bearing mice were treated under various conditions: control; NIR; PEG/Y-UCSZ + NIR, PEG/Y-UCSZ&DOX, and PEG/Y-UCSZ&DOX + NIR. The tumor size was recorded every two days after the initial treatment. As displayed in Fig. 7a, the relative tumor volumes (*V*/*V*_0_) are then drawn as a function of treated time. The tumor growth in mice exposed to the 980 nm laser was slightly inhibited over two weeks of treatment, possibly owing to the heat effect caused due to the absorbance of laser irradiation by hemoglobin. At the same time, the mice injected with the PEG/Y-UCSZ nanoparticles upon the radiation of 980 nm laser display a much smaller tumor size than the NIR-irradiated group, which may be caused by the PDT effect and photo-enhanced performance of PEG/Y-UCSZ. The tumor growth on PEG/Y-UCSZ&DOX treated mice is restrained to some extent after two weeks, which may be caused by the chemotherapy effect of DOX and a small Fenton effect. Especially, the mice treated with PEG/Y-UCSZ&DOX injection and NIR irradiation have the highest anticancer efficacy among all the groups, perhaps due to the superior therapy effect of the synergistic cooperation between PDT and chemotherapy. Besides, the weights of excised tumors corresponding to the mice belonging to the various treatment groups are displayed in Fig. 7b, directly demonstrating the high inhibition efficacy of the nanoparticles. To explain the potential toxicity *in vivo* more clearly, the pictures of representative mice and excised tumors also show that the tumor size treated with PEG/Y-UCSZ and NIR radiation was the smallest as shown in Fig. 7c, which illustrates that it has the greatest antitumor effect among different experimental groups. The experimental results are well consistent with the *in vitro* cell experiments. As shown in Fig. 7d, H&E stained photographs of tumor sections testify that the group treated with the PEG/Y-UCSZ injection and NIR irradiation shows the highest degree of tumor damage. H&E stained photographs of the major organs including heart, liver, spleen, lung and kidney are presented in Fig. S11.† The normal organs in five groups show no obvious damage, implying the high *in vivo* biocompatibility of PEG/Y-UCSZ&DOX.

## Conclusions

In a word, we successfully developed a multifunctional theranostic platform based on a yolk mesoporous nanoplatform (PEG/Y-UCSZ&DOX) to achieve photo-enhanced CDT, PDT, and chemotherapy with the inherent MRI/CT/UCL imaging-guidance. The yolk-structured PEG/Y-UCSZ was constructed in a single step by treating UCNPs@mSiO_2_ under facile hydrothermal conditions. The Yb^3+^/Tm^3+^-codoped UCNPs can be efficiently employed as a UV-Vis source, inducing the PDT effect and improving the efficiency of the Fenton reaction. As an effective photosensitizer, photogenerated electrons of ZnFe_2_O_4_ can convert H_2_O_2_ to ˙OH and photogenerated holes (h^+^) in the VB of ZnFe_2_O_4_, which can react with the surface-adsorbed H_2_O or OH^–^ to generate the highly reactive ˙OH after absorbing the UV energy from the UCNPs. What's more, the Fenton reaction can be observably enhanced upon the irradiation of a 980 nm laser, which significantly enhances the CDT effect. In addition, the yolk nanostructures with mesoporous shells and large cavities can provide more reactive sites for the Fenton reaction and photodynamic process, and are meanwhile suitable for loading DOX, achieving an enhanced anti-tumor effect. Such a multifunctional nanoplatform that integrates photo-enhanced PDT, CDT, and chemotherapy with the trimodal imaging shows tremendous potential in imaging-guided cancer therapy.

## Experimental section

### Reagents and materials

Zinc acetylacetonate (Zn(acac)_2_), iron(iii) acetylacetonate (Fe(acac)_3_), Tm_2_O_3_ (99.99%), Gd_2_O_3_ (99.99%), Yb_2_O_3_ (99.99%), sodium fluoride (NaF), 1-octadecene (ODE), oleic acid (OA), DA hydrochloride, 1-(3-dimethylaminopropyl)-3-ethylcarbodiimide hydrochloride (EDC), 2,7-dichlorofluorescein diacetate (DCFH-DA), methylene blue (MB), 2,2,6,6-tetramethylpiperidine (TEMP), 5,5-dimethyl-1-pyrroline-N-oxide (DMPO), doxorubicin (DOX), 1,3-diphenylisobenzofuran (DPBF), tetraethyl orthosilicate (TEOS), 3-4,5-dimethylthiazol-2-yl-2,5-diphenyl tetrazolium bromide (MTT), 4′,6-diamidino-2-phenylindole (DAPI), propidium iodide (PI), and mPEG5k–COOH (PEG, polyethylene glycol); hydrogen peroxide (H_2_O_2_), ammonium nitrate (NH_4_NO_3_), cetyltrimethyl ammonium bromide (CTAB), sodium trifluoroacetate (CF_3_COONa).

### Preparation of β-NaGdF_4_:Yb,Tm

The fabrication method of β-NaGdF_4_:Yb,Tm was based on previous literature reports. In short, 1 mmol of RE(oleate)_3_ (RE = 79% Gd + 20% Yb + 1% Tm) and a mixture of NaF (12 mmol) and 30 mL of OA and ODE (volume ratio is 1 : 1) were added successively to a three-necked flask. Then the mixed solution was stirred and progressively heated to 110 °C *in vacuo* for 30 min, later N_2_ was passed and the temperature was raised to 300 °C and maintained for 1.5 h. After that, the β-NaGdF_4_:Yb,Tm nanoparticles were obtained by centrifugation.

### Synthesis of NaGdF_4_:Yb,Tm@NaGdF_4_:Yb (denoted as UCNPs)

In general, 0.05 mmol of Yb(CF_3_COO)_3_, 0.45 mmol of Gd(CF_3_COO)_3_ and 1 mmol of CF_3_COONa were added into a reaction vessel with core nanoparticles, later OA (15 mL) and ODE (15 mL) were added. The admixture was progressively heated to 120 °C with stirring for 1 h. After N_2_ was allowed to flow through the mixture, the mixture was heated to 310 °C and maintained at that temperature for 1 h. After that, the UCNPs were centrifuged and dispersed in cyclohexane..

### Synthesis of UCNPs@mSiO_2_ (abbreviated as UCS)

In short, 0.1 g of CTAB and 20 mL of water were added in a beaker and sonicated to obtain a clear solution; after that 2 mL of UCNPs was heated for about 10 minutes, and then stirred overnight at room temperature until the solution was clear. After that, 40 mL of water, 6 mL of absolute ethanol and 300 μL NaOH (2 M) were added to each cup. The mixture was heated on a water bath, and after maintaining at 70 °C for a few min, 200 μL of orthosilicate was added dropwise and stirred for 10 min. The above-mentioned mixed solution was added to ethanol to rapidly cool down and was then washed with ethanol several times. In order to clear away CTAB, 50 mL of the above mixture solution and 0.3 g of NH_4_NO_3_ were added to a three-necked flask, and allowed to react in a water bath at 60 °C for 2 h, followed by centrifugation with ethanol and water, respectively. Ultimately, the samples were obtained.

### Synthesis of yolk-structured mesoporous ZnFe_2_O_4_ coated UCS (Y-UCSZ)

20 mg of UCNPs@mSiO_2_ nanoparticles were uniformly scattered in 40 mL water, then 0.2 mmol Zn(acac)_2_ and 0.4 mmol Fe(acac)_3_ were added to the UCNPs@mSiO_2_ nanosphere suspension. After stirring for half an hour, the mixed solution was diverted to a Teflon-lined autoclave and maintained at 180 °C for 12 h, afterwards the solution was cleaned with DI and ethanol several times by centrifugation. Finally, the Y-UCSZ samples were dried at 60 °C in a vacuum oven overnight.

### Synthesis of PEGylated Y-UCSZ (PEG/Y-UCSZ) nanoparticles

DA hydrochloride (60 mg) and Y-UCSZ (20 mg) were added to DI water (30 mL) and stirred at normal temperature for 12 h. Later, the DA modified Y-UCSZ (DA/Y-UCSZ) was obtained and collected by centrifugation. The product was rinsed with ethanol and deionized water several times in order to wash away the unreacted DA hydrochloride. After that, the obtained DA/Y-UCSZ was dispersed in DI water (20 mL), and then 10 mL mPEG–COOH solution was added to Y-UCSZ–DA solution under ultrasonication for 30 min. Then 20 mg EDC was stirred for 12 h, the prepared PEG/Y-UCSZ was collected by centrifugation and cleaned with water several times.

### Extracellular ^1^O_2_ detection

DPBF was used to detect the generation of extracellular ^1^O_2_. Typically, 1 mL of PEG/Y-UCSZ solution (0.5 mg mL^–1^) was mixed with 1 mL of ethanol (containing 1 mg of DPBF). The admixture was irradiated by 980 nm light (0.8 W cm^–2^) in a dark room for different periods (0, 3, 5, 7, and 10 min). After that the supernatant was prepared for UV-Vis measurements.

### Detection of ˙OH

Methylene blue (MB), as an indicator, was chosen to detect the production of ˙OH. The PEG/Y-UCSZ nanoparticles (1 mg) were added into MB solution (10 mg L^–1^). Afterwards, H_2_O_2_ (0, 10, 20, 30, 50 μM) was mixed under the irradiation of a 980 nm laser (0.8 W cm^–2^) for various periods (0, 3, 5, 7 and 10 min), then MB solution was centrifuged to wash away PEG/Y-UCSZ nanoparticles. The absorbance was detected by UV-Vis at 663 nm.

### Detection of intracellular ROS production

DCFH-DA was used to detect the intracellular ROS generation ability of the PEG/Y-UCSZ. HeLa cells were cultivated in a six-well plate for 12 h, after that 1 mL of PEG/Y-UCSZ was incubated for 3 h, later DCFH-DA was incubated for 10 min and washed with PBS several times. After irradiation with a 980 nm laser for 1, 3, 5 and 7 min, the fluorescence images were obtained at the wavelength of 488 nm.

### DOX loading and releasing test

PEG/Y-UCSZ (20 mg) was scattered into a PBS solution of DOX (20 mL, 0.5 mg mL^–1^) under stirring in aphotic surroundings. DOX-loaded PEG/Y-UCSZ (PEG/Y-UCSZ&DOX) nanoparticles were collected by centrifugation; the supernatant was collected to determine the DOX loading rate using a UV-Vis instrument. The sediment was reserved for further DOX emancipation procedure. PBS was added into a water bath kettle at 37 °C with magnetic stirring, and later the supernatant was collected for UV-Vis analysis. At different time intervals, the emancipation procedure was duplicated in PBS solutions at various pH values (7.4, 6.5, and 5.5) or temperatures (25 °C, 37 °C, and 50 °C).

### Characterization

UV-Vis absorption spectra were measured by using a TU-1901 dual beam UV-Vis spectrophotometer. Transmission electron microscopy (TEM) micrographs were obtained using a FEI Tecnai G^2^ S-twin transmission electron microscope. The sample for the X-ray diffraction (XRD) assay was obtained by depositing the product solution on glass slides and vacuum drying at 80 °C. Fourier-transform infrared (FT-IR) spectra were recorded on a Vertex Perkin-Elmer 580BIR spectrophotometer (Bruker). Up conversion emission spectra were recorded on an Edinburgh FLS 980 apparatus, from 400 to 700 nm, using a 980 nm laser diode module as the irradiation source. The N_2_ adsorption–desorption isotherm and pore-size distribution were observed on a Micromeritics Tristar 3000 instrument.

### *In vitro T*_2_-weighted MR imaging

The *in vitro* and *in vivo* MR imaging experiments were performed using a 0.5 T MRI magnet. The PEG/Y-UCSZ was scattered in water at different concentrations of Fe and Gd. The *T*_2_ measurements were conducted before and after injection of PEG/Y-UCSZ (100 μL, 4 × 10^–3^ M). Finally, the *r*_2_ relativity values were determined by the curve fitting of 1/*T*_2_ relaxation time (s^–1^) *versus* the total concentration of Fe and Gd (mM).

### *In vitro* and *in vivo* X-ray CT imaging

A Philips 64-slice CT scanner at a voltage of 120 kV was employed to perform the *in vitro* CT imaging experiments. The sample was dispersed in PBS at various concentrations (0, 0.75, 1.5, 3, 6, 12 mg mL^–1^) and later placed normatively for CT imaging assay. For *in vivo* X-ray CT imaging, two female mice were first anesthetized with 10% chloral hydrate (0.03 mL g^–1^ of mouse). After that, one mouse was injected 100 μL of PEG/Y-UCSZ saline solution (4 mg mL^–1^), and another mouse was treated with saline as the control. Finally, the results were acquired after scanning.

### *In vitro* cellular uptake and UCL microscopy observation

The cellular uptake process of PEG/Y-UCSZ on HeLa cells was investigated using a confocal laser scanning microscope (CLSM). HeLa cells were cultivated in a 6-well plate and incubated overnight. Thereafter, 1 mL of PEG/Y-UCSZ&DOX (1 mg mL^–1^) was added to the wells and respectively cultured for 0.5, 1, and 3 h. Later, the cells were cleaned with PBS several times and dyed using DAPI for 10 min. Then 1 mL of glutaraldehyde (2.5%) was employed to fix the cells for 10 min, and washed with PBS. Finally, the fluorescence images of cells were recorded employing a Leica TCS SP8 instrument. As for UCL microscopy observation, the slides were obtained using the same procedure except that the slides were treated with a radiation of 980 nm laser and observed employing an inverted fluorescence microscope (Nikon It-S).

### *In vitro* cytotoxicity

A representative MTT survey was employed to assess the *in vitro* cytotoxicity. To evaluate the cytotoxicity of as-prepared samples against cancer cells, HeLa cells were cultivated in a 96-well plate and incubated in the incubator (37 °C, 5% CO_2_) overnight. PEG/Y-UCSZ and PEG/Y-UCSZ&DOX were scattered into the culture media at the concentrations of 0, 15.6, 31.2, 62.5, 125, 250, and 500 mg mL^–1^, and later the cells were treated with the control, NIR irradiation, PEG/Y-UCSZ + NIR, PEG/Y-UCSZ&DOX, and PEG/Y-UCSZ&DOX + NIR (pump power: 0.8 W cm^–2^), respectively. The samples were cultivated for 4 h to accomplish the cell uptake, after that the irradiation was carried out for 5 min. Afterwards, 20 μL of MTT solution (5 mg mL^–1^) was added into each well and incubated for 4 h, then 150 μL of DMSO was added and the absorbance was recorded at 490 nm for calculation. The *in vitro* biocompatibility of PEG/Y-UCSZ with L929 fibroblast cells was also assessed by a similar MTT assay. The concentrations of the samples were 15.6, 31.2, 62.5, 125, 250, and 500 μg mL^–1^.

### *In vivo* toxicity

Female Balb/c mice (20 to 25 g) were purchased from the Harbin Veterinary Research Institute, Chinese Academy of Agricultural Sciences (Harbin, China), and all mouse experiments were performed in compliance with the criteria of The National Regulation of China for Care and Use of Laboratory Animals and approved by the Harbin Science and Technology Bureau. The tumor xenograft was implanted into the left axilla of each mouse (16–20 g). When the tumor size grew to 6–9 mm approximately, all mice were stochastically divided into five groups (*n* = 5 per group). The five groups of mice were treated respectively with: control; NIR; PEG/Y-UCSZ&DOX; PEG/Y-UCSZ + NIR; PEG/Y-UCSZ&DOX + NIR. For the procedure of NIR irradiation, the tumor site was irradiated with a 980 nm laser for 5 min after nanoparticle injection for 4 h (0.8 W cm^–2^). The tumor sizes were recorded every 2 days during the process of treatment. The weights of excised tumors corresponding to the mice from various treatment groups were obtained at the end of the treatment. Tumor volume (mm^3^) was calculated according to the equation *V* = *lw*^2^/2, in which *l* and *w* represent the length and width of the tumor.

### Histological examination

The histological analysis was implemented after two weeks of therapy. Tissues in the organs of heart, lung, liver, spleen, kidney, and tumor of the representative mice in five groups were cut off. After that, the excised tissues were successively dehydrated using buffered formalin, ethanol at different concentrations, and xylene. Finally, the above dehydrated tissues were embedded in liquid paraffin, and sliced for hematoxylin and eosin (H&E) staining. The final stained sections were investigated employing an optical microscope.

## Conflicts of interest

There are no conflicts to declare.

## Supplementary Material

Supplementary information
