# Supplementary material for: Upconversion-mediated ZnFe2O4 nanoplatform for NIR-enhanced chemodynamic and photodynamic therapy
Source: Chem Sci. 2019 Mar 6;10(15):4259–71. doi: 10.1039/c9sc00387h (PMC6471739; doi:10.1039/c9sc00387h)
Supplement: Supplementary file 1 [file SC-010-C9SC00387H-s001.pdf]

Electronic Supplementary Material (ESI) for Chemical Science.
This journal is © The Royal Society of Chemistry 2019

Electronic Supplementary Information

Upconversion-mediated $ZnFe_2O_4$ nanoplatform for NIR-enhanced chemodynamic and photodynamic therapy

Shuming Dong,[a] Jiating Xu,[a] Tao Jia,[a] Mengshu Xu,[a] Chongna Zhong,[a] Guixin Yang,[a] Jiarong Li,[a] Dan Yang,[a] Fei He,[a] Shili Gai,*[a] Piaoping Yang,*[a] and Jun Lin *[b]

[a] Key Laboratory of Superlight Materials and Surface Technology, Ministry of Education, College of Material Sciences and Chemical Engineering, Harbin Engineering University, Harbin, 150001, P. R. China

[b] State Key Laboratory of Rare Earth Resource Utilization, Changchun Institute of Applied Chemistry, Chinese Academy of Sciences, Changchun 130021, P. R. China

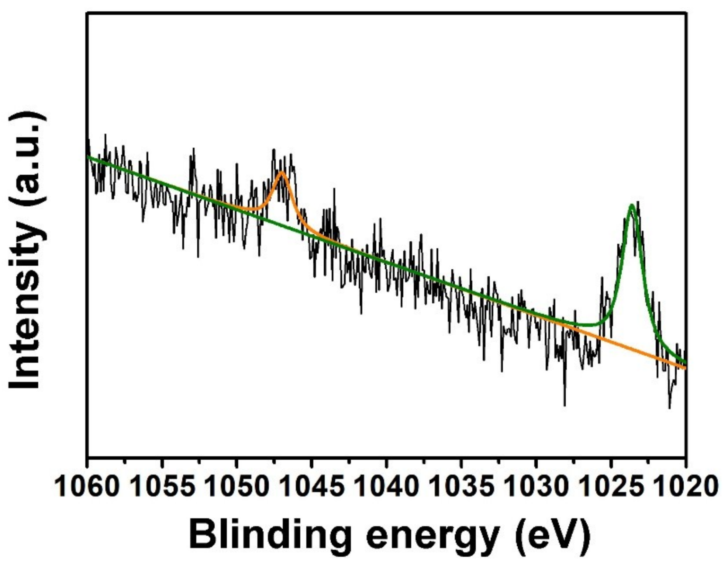

Fig. S1 XPS spectrum of Zn 2p in PEG/Y-UCSZ.

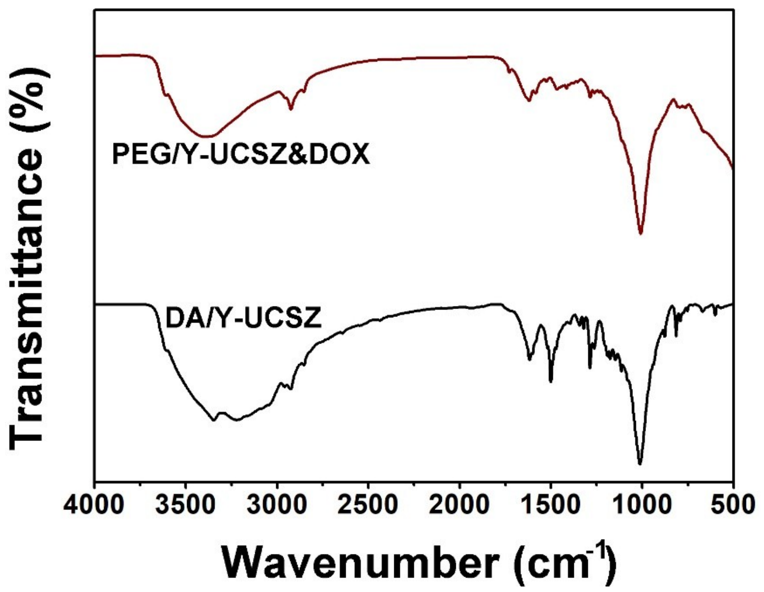

Fig. S2 FT-IR spectra of the DA/Y-UCSZ and DOX loaded PEG/Y-UCSZ samples.

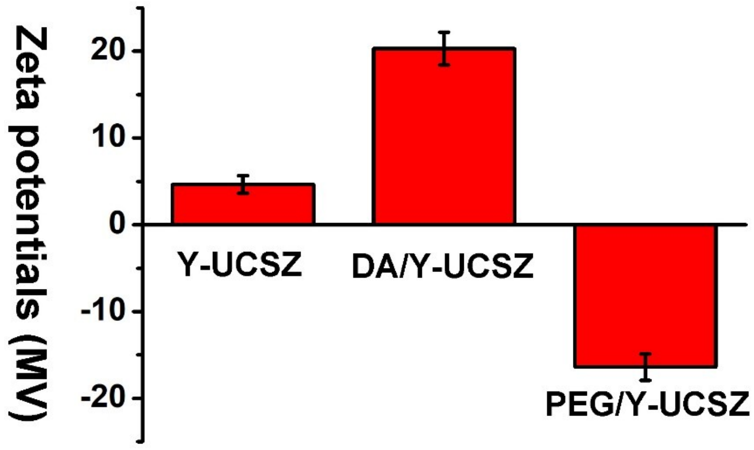

Fig. S3 The zeta potentials of the Y-UCSZ, DA/Y-UCSZ and PEG/Y-UCSZ samples. Date are presented as means ± standard deviation (s. d.) (n = 3)

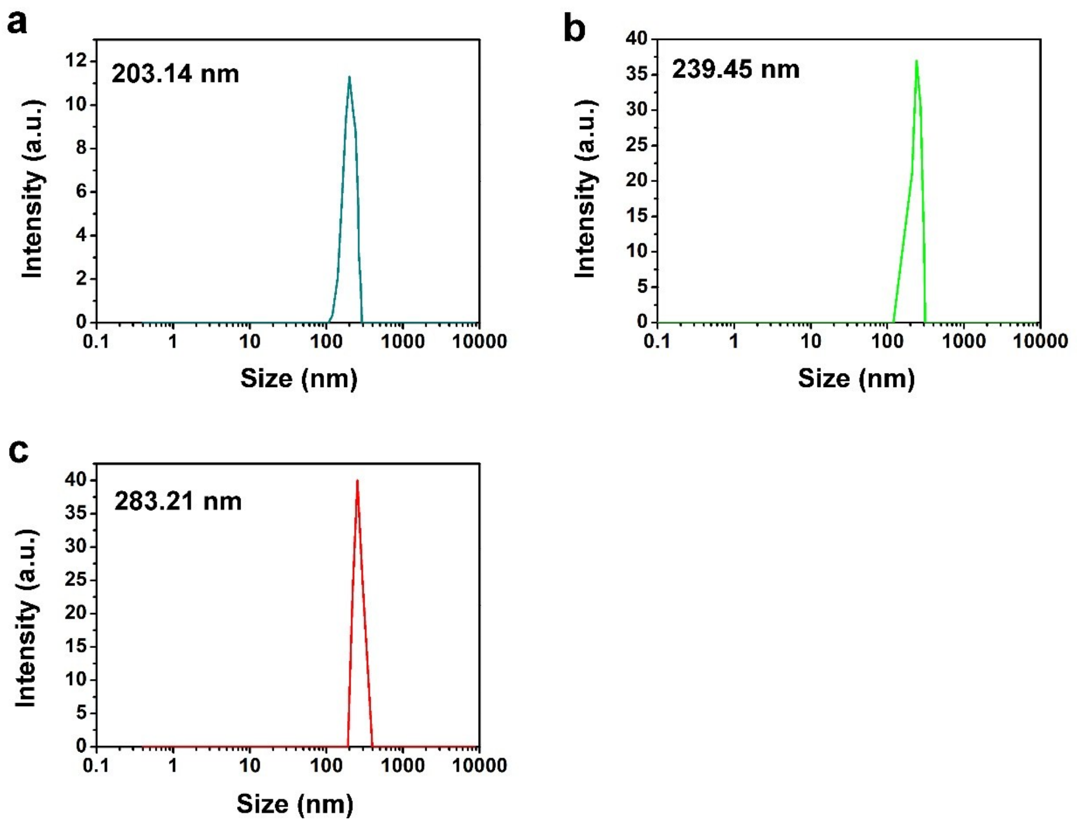

Fig. S4 DLS measurements of Y-UCSZ (a), DA-Y-UCSZ (b), and PEG/Y-UCSZ (c).

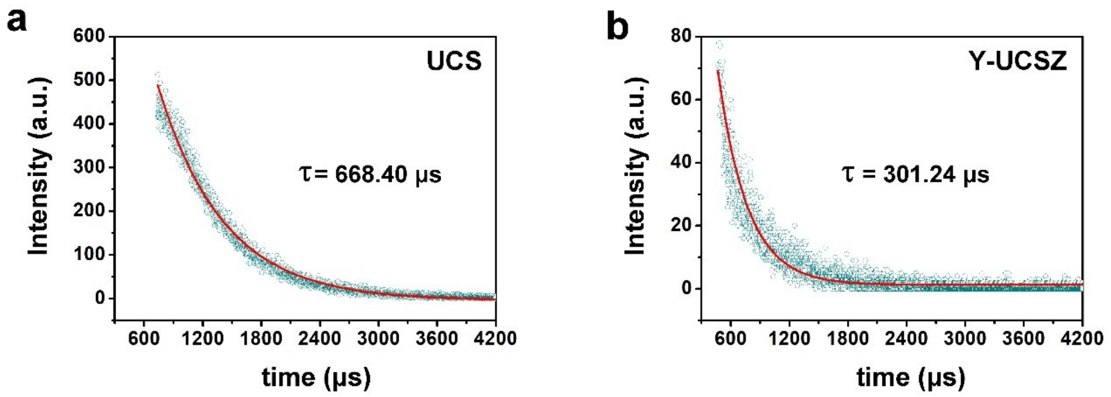

Fig. S5 Decay curves for $^1G_4$–$^3H_6$ emission (475 nm) of Tm^{3+} in UCS (a) and Y-UCSZ (b).

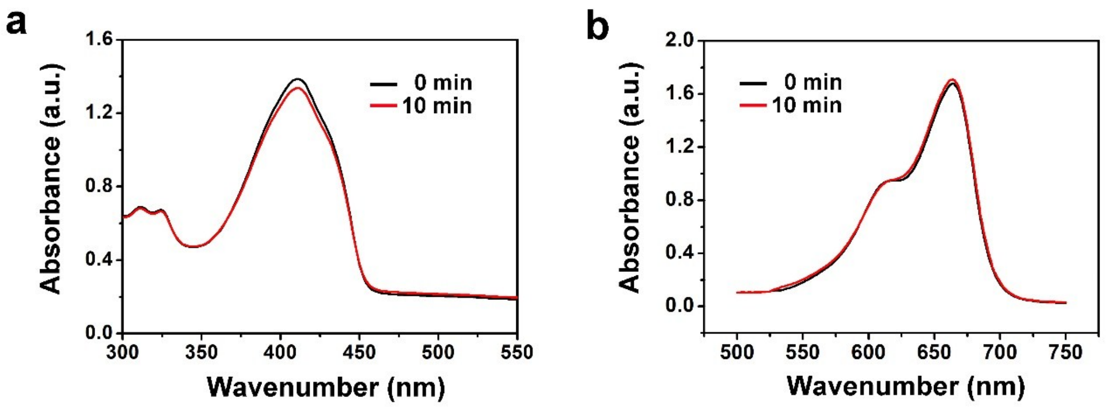

Fig. S6 Absorption spectra of DPBF (a) and MB (b) solutions under 980 nm laser irradiation.

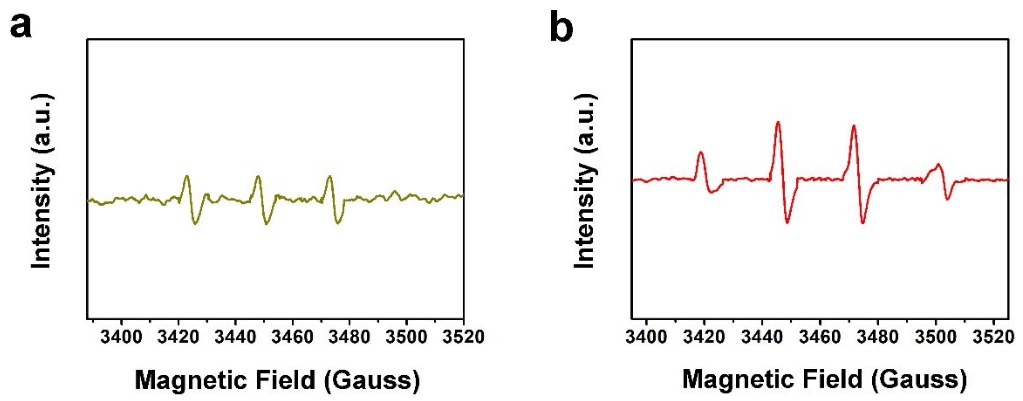

Fig. S7 EPR spectra of $^1O_2$ (a) and $\cdot OH$ (b) in the PEG/Y-UCSZ aqueous solution (5 mg mL^{-1}) with 980 nm laser irradiation in the presence of the spin trap TEMP and DMPO.

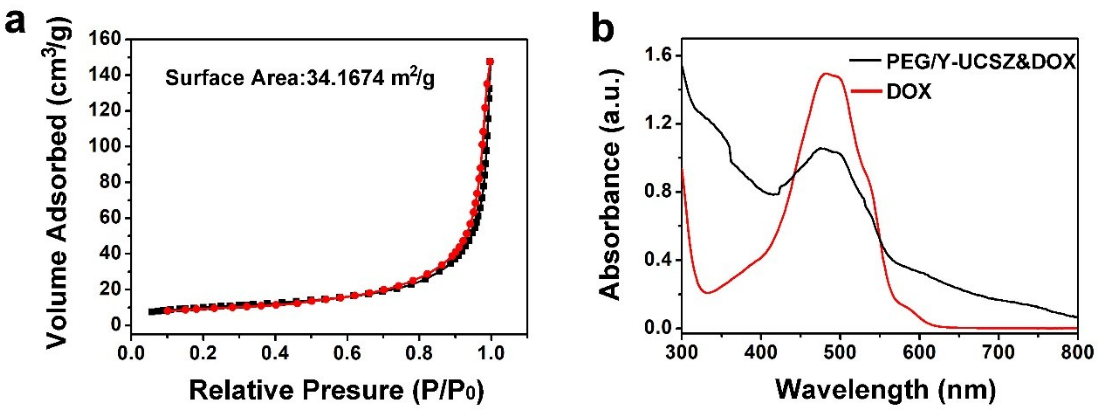

Fig. S8 N_2 absorption-desorption isotherm of DOX loaded PEG/Y-UCSZ (a). UV-Vis absorption spectra of the DOX molecules and the final PEG/Y-UCSZ&DOX (b).

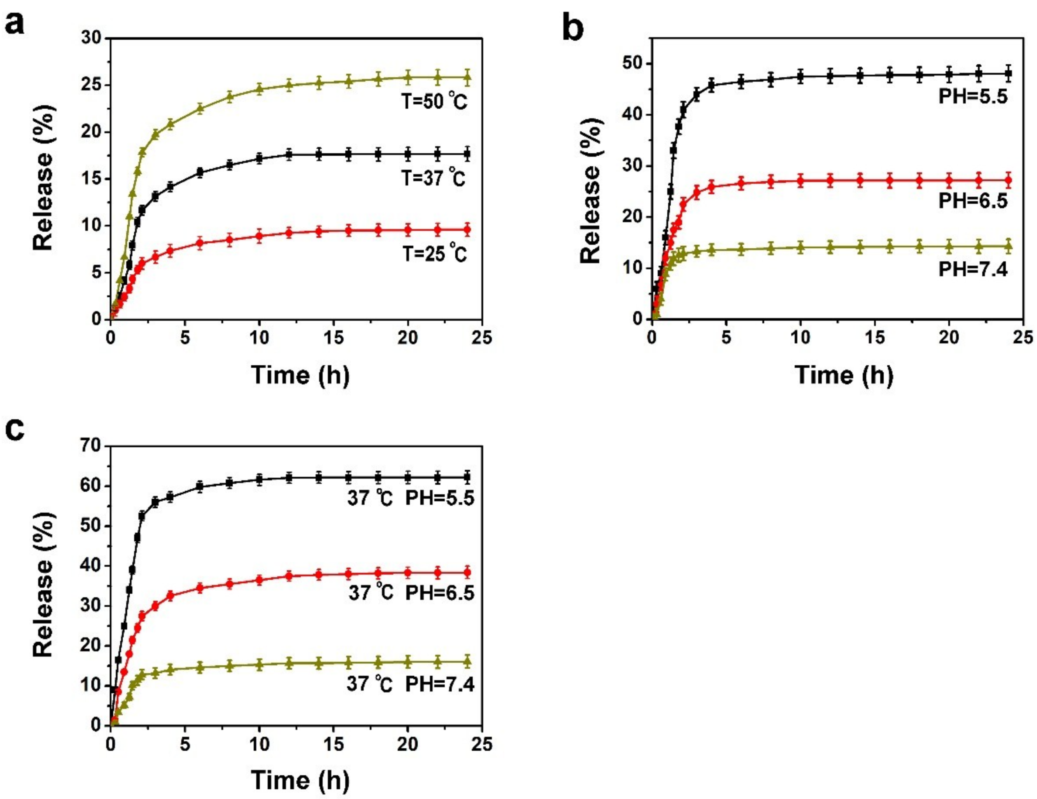

Fig. S9 DOX release efficiency from PEG/Y-UCSZ&DOX in PBS at varied temperatures (pH = 7.4) (a), pH values (T = 37 °C) (b), and combined acidic and temperature conditions (c).

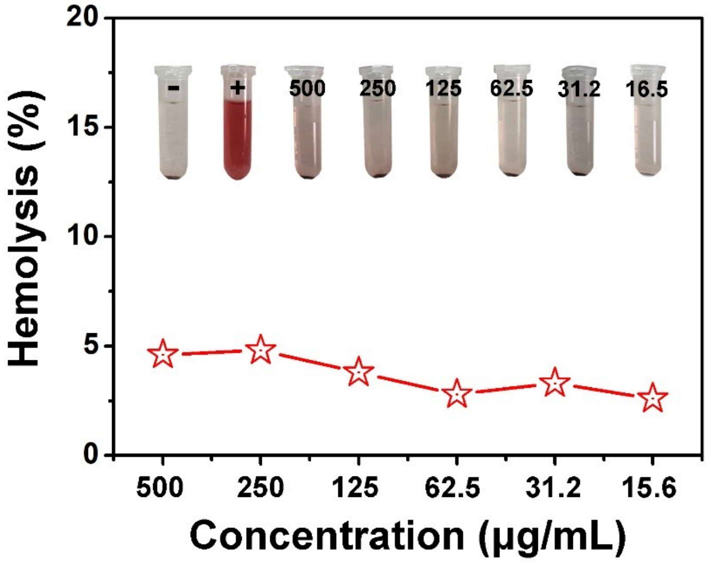

Fig. S10 The hemolytic percentage of PEG/Y-UCSZ to human red blood cells.

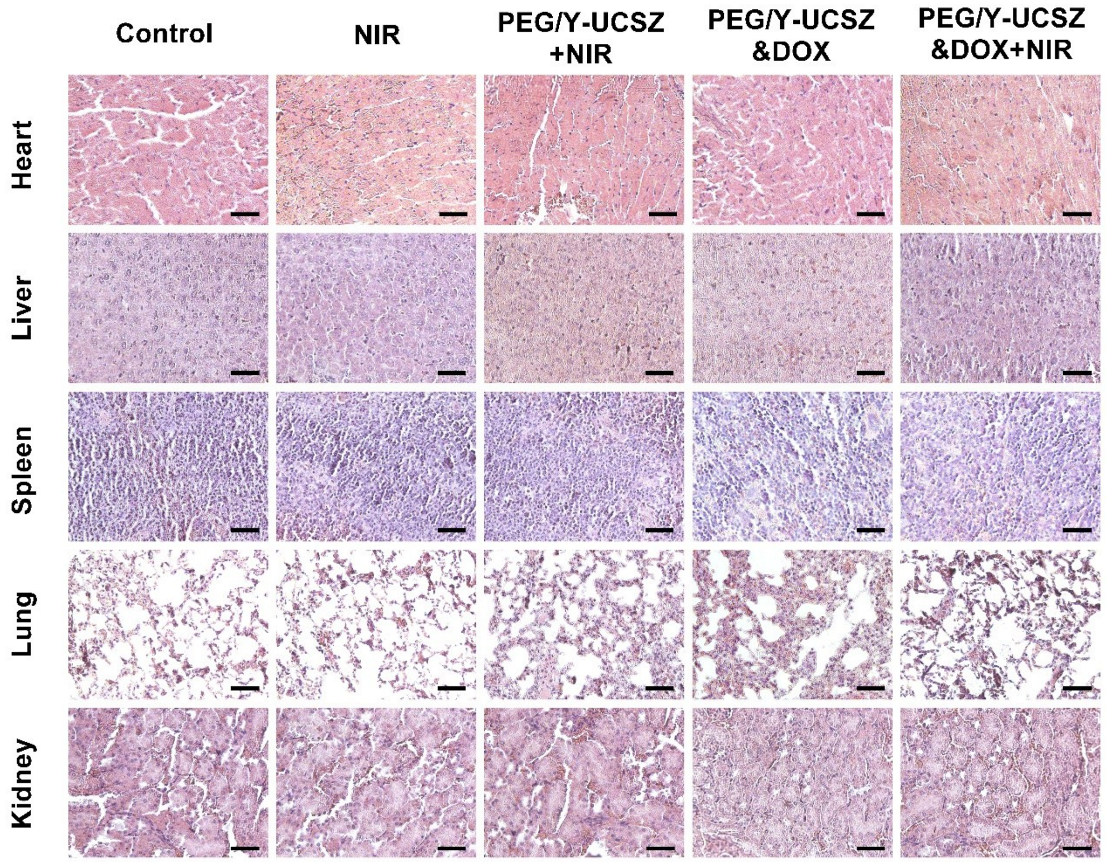

Fig. S11 H&E stained images of liver, lung, kidney, heart and spleen achieved from different groups after 14 days treatment. Scale bar: 50 μm.
